# Organic Farming Favors *phoD*-Harboring Rhizospheric Bacterial Community and Alkaline Phosphatase Activity in Tropical Agroecosystem

**DOI:** 10.3390/plants12051068

**Published:** 2023-02-27

**Authors:** Neha, Yashpal Bhardwaj, Bhaskar Reddy, Suresh Kumar Dubey

**Affiliations:** 1 Centre of Advanced Study in Botany, Institute of Science, Banaras Hindu University, Varanasi 221005, India; 2 Survey of Medicinal Plant Unit, Regional Ayurveda Research Institute, Itanagar 791111, India; 3 Virus Research and Diagnostic Laboratory, Government Institute of Medical Sciences, Greater Noida 201310, India

**Keywords:** alkaline phosphatase activity, available phosphorous, *phoD* abundance, farming practice, high-throughput sequencing

## Abstract

The bacteria harboring *phoD* encodes alkaline phosphatase (ALP), a secretory enzyme that hydrolyzes organic phosphorous (P) to a usable form in the soil. The impact of farming practices and crop types on *phoD* bacterial abundance and diversity in tropical agroecosystems is largely unknown. In this research, the aim was to study the effect of farming practices (organic vs. conventional) and crop types on the *phoD*-harboring bacterial community. A high-throughput amplicon (*phoD* gene) sequencing method was employed for the assessment of bacterial diversity and qPCR for *phoD* gene abundance. Outcomes revealed that soils treated for organic farming have high observed OTUs, ALP activity, and *phoD* population than soils managed under conventional farming with the trend of maize > chickpea > mustard > soybean vegetated soils. The relative abundance of *Rhizobiales* exhibited dominance. *Ensifer*, *Bradyrhizobium*, *Streptomyces*, and *Pseudomonas* were observed as dominant genera in both farming practices. Overall, the study demonstrated that organic farming practice favors the ALP activity, *phoD* abundance, and OTU richness which varied across crop types with maize crops showing the highest OTUs followed by chickpea, mustard, and least in soybean cropping.

## 1. Introduction

Phosphorous is one of the most important macronutrients essential for the growth and development of plants. Regardless of its abundance in soils, the availability of phosphorous is very limited in the terrestrial environment. Generally, two forms of P are present in the soil, organic and inorganic forms; however, only inorganic orthophosphate (PO_4_^3−^) ions in the soil solution are easily accessible for plant use [1]. A huge amount of P fertilizers is utilized in agriculture as mineral P fertilizers and organic fertilizers (compost/manure) to maintain crop productivity. Soon after its application in the soil, a portion of inorganic P is readily used by the plants and microbes, whereas the residual P is immobilized as an insoluble form in the soil. Microbes play a vital role in recycling phosphorous from a recalcitrant unavailable form of P by solubilization and mineralization of inorganic and organic forms of P, respectively [2]. Inorganic P in soil mainly comprises minerals, such as apatite, oxyapatite, and hydroxyapatite, which are poorly soluble and assimilable. The inorganic P is solubilized by soil microbes by excreting organic acids [3]. The organic P mineralization is through the enzymatic process by the P-hydrolyzing extracellular enzyme phosphatases [1,2] which comprise phosphomonoesterases, phosphodiesterases, phosphotriesterases, etc. The phosphomonoesterases are composed of alkaline and acid phosphatases, nucleotidases, and phytases. Activities of both alkaline and acid phosphatases in soil have been analyzed to assess the organic phosphorous mineralization to inorganic phosphorous form [4]. Alkaline phosphatases (ALP) primarily originate from soil microorganisms, especially bacteria, which are involved in the hydrolysis of organic phosphorous [4]. To date, three homologous ALP encoding gene families have been identified, namely, *phoA*, *phoD*, and *phoX*, as a component of Pho regulon [5]. According to metagenomics datasets, based on the sequence similarity, *phoD* is the most frequently available gene than *phoA* and *phoX*. *phoD*-harboring bacteria have been widely identified and ubiquitously distributed among terrestrial and aquatic ecosystems [6]. Hence, *phoD* is considered a good biomarker to bestow an understanding of P transformation in an agroecosystem. Some studies showed a negative correlation between the activity of the ALP enzyme and available phosphorous [7,8]. There are reports that bacteria enhance the production of ALP during scarcity of P by upregulating the expression of functional gene encoding phosphatase enzyme [4]. Moreover, some studies reported a positive correlation between alkaline phosphatase activity and *phoD* copy number [9,10,11]. However, more evaluation of the origin/source of *phoD* is required to amplify our knowledge to understand the relationship between the potential activity of alkaline phosphatase and available P in the soil [12].

Researchers have shown the shift in *phoD*-gene-containing bacterial population under different environmental sites and experimental conditions, including fertilizer management practices in agriculture [6,8,11], soil pH [12], amendment of organic matter [11,13,14], and extreme environments [15]. Tan et al. studied that in pasture soil phosphorous fertilizer incorporation enhanced the *phoD* community [5]. Xie et al. carried out a field experiment in the temperate monsoon climate of China to study the effect of fertilization treatment, crop rotation, and wheat varieties on functional communities of the rhizosphere associated with P cycling [16]. They found that crop rotation changed the community composition of bacteria having *phoD* genes in the wheat rhizosphere, whereas fertilization management had no effect. A negative correlation was seen between the *phoD* abundance and available P, P uptake, and wheat biomass. In another study, Fraser et al. reported that *phoD* copy number correlated positively with ALP activity in manure- and mineral-phosphorous-treated soils [9]. Results from the Illumina high-throughput approach revealed that the response of *phoD* bacteria to the P status of the soil is asynchronous, and nitrogen, carbon, and phosphorus soil stoichiometric ratios were the most dominant regulatory parameters for the *phoD* bacterial population in soils of Inner Mongolia [17]. Most of the studies regarding ALP activity and *phoD*-containing bacterial diversity to date have been carried out in the temperate and subtropical zones. In a recent study, Hegyi et al. reported that *Actinobacteria*, *Acidobacteria*, *Cholroflexi*, *Firmicutes*, and *Proteobacteria* are the superior phyla analyzed through next-generation sequencing of the 16S *rRNA* gene in the agricultural soil of Vietnam [18]. The study showed a positive correlation between soil phosphatase activities and soil organic C and also between acid phosphatase and total P. Similarly, a significant positive relationship was found between the abundance of the *phoD* gene and the diversity of the bacterial community of soil. There are still very limited studies with reference to the influence of various factors on *phoD* soil bacterial abundance and community and ALP activities, especially in an agricultural system with different fertilizer management practices.

However, as per the reviewed literature, information regarding the *phoD*-containing bacterial distribution, diversity, and community composition is still lacking, particularly in the Indian tropical agroecosystem. To procure a better understanding of the process involved through which bacteria participate in P turnover, there is a need for more studies focusing on the *phoD* bacterial population in the soil. In this study, we employed a high-throughput targeted amplicon sequencing to investigate the *phoD*-containing bacterial population in response to different farming practices vegetated with varied crops. In the present study, we hypothesized that the impact of farming practices (organic vs. conventional) and crop types would alter the abundance and composition of *phoD*-gene-harboring bacterial communities and soil alkaline phosphatase activity. The pivotal goal of this study was to (1) evaluate the influence of farming practices (organic vs. conventional) on ALP enzyme activity, *phoD* bacterial abundance, and diversity in soil under the influence of different crops and (2) assess the relationship between the ALP activity and *phoD* abundance and community.

## 2. Results

### 2.1. Soil Variables and Crop Biomass

**Figure 1:**
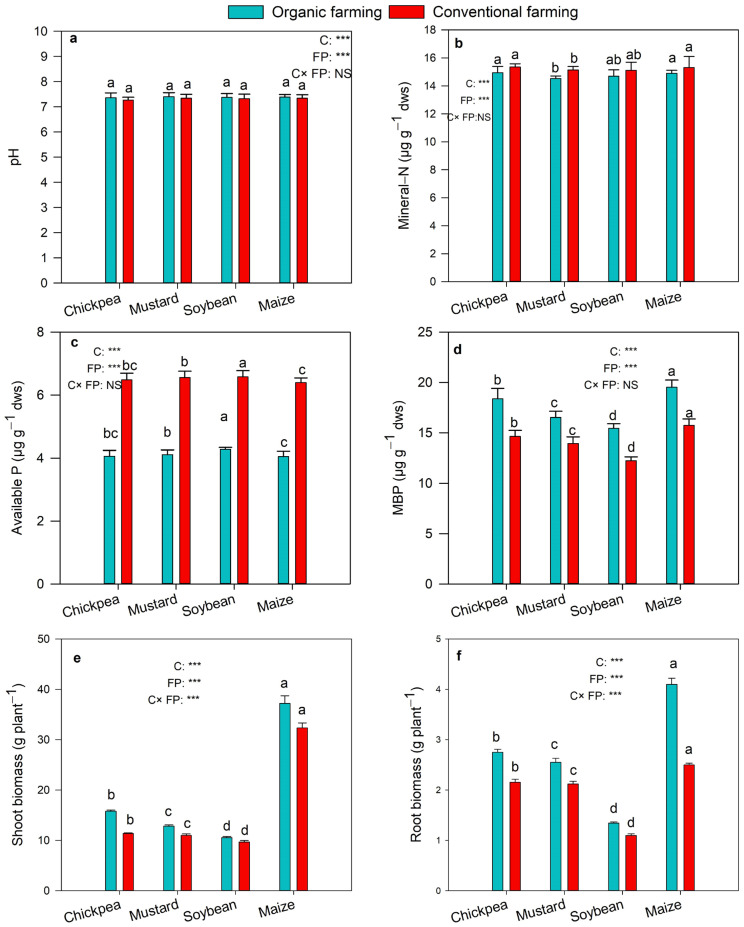
pH value (**a**), content of mineral-N (**b**), available P (**c**), MBP (**d**), shoot biomass (**e**), and root biomass (**f**) in organic and conventional farming. Different lowercases indicate significant differences (Tukey’s post hoc test) between crops in the same farming practice (*p* < 0.05). The crops (C) and farming practice (FP) specify the results of MANOVA indicating *p*-value at different significance levels, *** *p* < 0.001, NS: not significant). The values are two-year average mean ± SD. MBP, microbial biomass P; ALP, alkaline phosphatase.

The value of pH of the soil was significantly increased in soils treated for organic farming than the conventional (Figure 1a). Mineral-N content was highest in conventional farming (Figure 1b). A significant increase in available P was detected in conventional farming compared to organic treatments, and the effect of crops on the available P was also significant (Figure 1c; *p* < 0.001). The microbial biomass P (MBP) showed a significantly higher value in organic farming, and the effect of the crop on microbial biomass P was also significant (Figure 1d; *p* < 0.001). The root and shoot biomass were observed highest in organic farming practice, and the effect of crops and farming practice on the root and shoot biomass was also significant (Figure 1e,f; *p* < 0.001).

### 2.2. Soil Alkaline Phosphatase (ALP) and Abundance of phoD Gene

**Figure 2:**
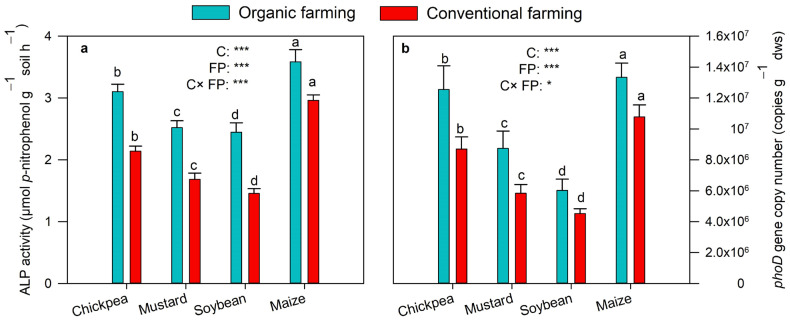
ALP activity (**a**) and *phoD* gene copy number (**b**) in organic and conventional farming. Different lower cases indicate significant differences (Tukey’s post hoc test) between crops in the same farming practice (*p* < 0.05). The crops (C) and farming practice (FP) specify the results of MANOVA indicating *p*-value at different significance levels (* *p* < 0.05, *** *p* < 0.001). The values are two-year average mean ± SD. MBP, microbial biomass P; ALP, alkaline phosphatase.

Variations in soil ALP activity and *phoD* gene copy number in rhizosphere soils of crops vegetated in both farming fields are depicted in Figure 2a,b. The ALP activity ranged from 2.45–3.59 µmol g^−1^soil h^−1^ in organic to 1.46–2.96 µmol g^−1^soil h^−1^ in conventional farming soils. Within the crops, maize showed the highest ALP activity and soybean the least (Figure 2a). MANOVA showed a significant (*p* < 0.001) effect of farming practices and crops on ALP enzyme activity. Tukey’s HSD test indicated significant variation in ALP activity between different crops in the same farming practice. qPCR analysis showed that, compared to conventional farming (4.5 × 10^6^–1.1 × 10^7^ copies g^−1^ dws), the *phoD* population was substantially higher among organic farming, which ranged from 6 × 10^6^ to 1.3 × 10^7^ copies g^−1^ dws (Figure 2b). The abundance of *phoD* gene copy was highest in soil samples of maize crops, followed by chickpea, mustard, and soybean soils. The MANOVA result showed a significant (*p* < 0.001) effect of farming practices and types of crops on the abundance of *phoD* gene copy. Tukey’s HSD test suggested *phoD* abundance to differ significantly between different crops.

### 2.3. phoD-Gene-Containing Bacterial Community and Relationship with Soil Parameters

Initially, a total of 674,076 raw reads were generated from *phoD* amplicon 2 × 300 pair-end sequencing, with an average of 84,260 reads per sample. The stringent poor-quality filtration resulted in a total of 254,895 high-quality reads subjected for downstream analyses. To compare *phoD*-gene-harboring bacterial community among different samples, rarefaction of sequences/reads was performed to obtain the OTUs assigned by an equal number of sequences. We rarified the sequences at minimum library size to 2629 reads for each sample. The *phoD* OTUs ranged between 346 and 672 with ~457 average OTUs per sample (Table 1). The observed OTUs composition of *phoD* gene bacteria was greater in soils treated for organic farming than the conventional one (Table 1). The highest OTUs were reported in soils of maize crop under organic farming, followed by chickpea-, mustard, and soybean-planted soils.

We estimated the Pearson correlation coefficient between the diversity indices, soil parameters, and *phoD* copy number (Table 2). The Pearson correlation showed a significant (*p* < 0.01) positive correlation between OTUs richness, Shannon and Simpson index with that of *phoD* abundance, and ALP activity of soil (Table 2).

**Figure 3:**
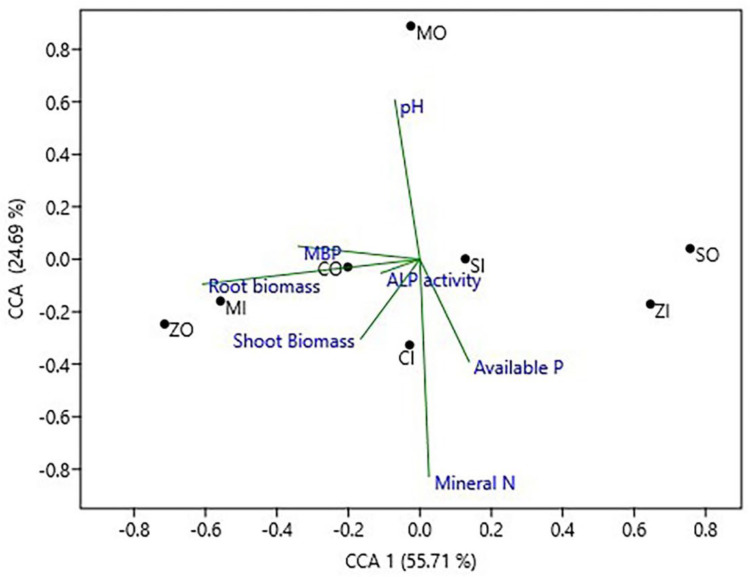
Ordination plot of CCA (Canonical Correspondence Analysis) to exhibit the correlation between soil variables and *phoD* encoding community structure. CO, chickpea organic farming; CI, chickpea conventional farming; MO, mustard organic farming; MI, mustard conventional farming; SO, soybean organic farming; SI, soybean conventional farming; ZO, maize organic farming; ZI, maize conventional farming; MBP, microbial biomass P.

The results of CCA (canonical correspondence analysis) showed that the structure of *phoD* bacterial community significantly correlated with ALP activity, MBP, and available P and to a lesser extent with pH and mineral-N (Figure 3). The *phoD* community structure in different farming practices in all four crops changed along the first axis. The soil pH and mineral-N showed a strong correlation with the CCA2 axis that governs 24.69% of the overall variance in the *phoD* community.

### 2.4. Relative Abundance of phoD Bacterial Community

**Figure 4:**
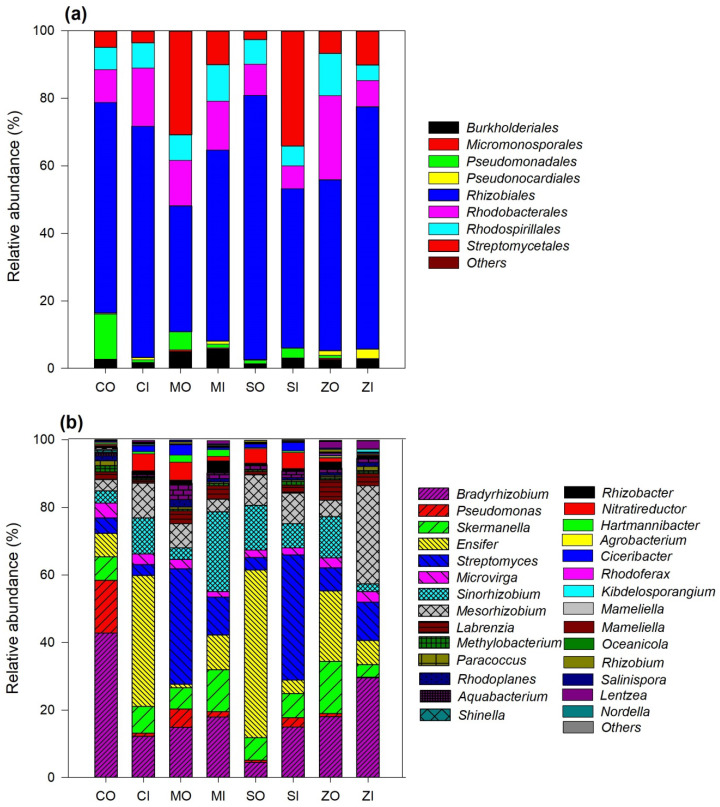
(**a**) Relative abundance of *phoD*-harboring bacterial communities under organic and conventional farming practices and different crops at the order level. (**b**) Relative abundance of *phoD*-harboring bacterial communities under organic and conventional farming practices and different crops at the genera level. CO, chickpea organic farming; CI, chickpea conventional farming; MO, mustard organic farming; MI, mustard conventional farming; SO, soybean organic farming; SI, soybean conventional farming; ZO, maize organic farming; ZI, maize conventional farming.

All the *phoD* reads were affiliated with the phyla Proteobacteria and Actinobacteria. Proteobacteria was the dominant phylum accounting for 65.24–94.57% of all the sequences. These OTUs were classified into eight bacterial orders that include Rhizobiales, Streptomycetales, Pseudomonadales, Rhodobacteriales, Rhodospirallales, Burkholderiales, Pseudonocardiales, and Micromonosporales. The Rhizobiales were dominant among all samples and accounted for 37.28–78.27% (Figure 4a). The *phoD*-containing bacterial order varied among farming practice and crops, but no particular trend was observed. A total of 29 genera were identified, among which the dominant genera present were *Bradyrhizobium* (4.41–42.73%), *Ensifer* (1.04–49.65%), *Streptomyces* (3.29–37.08%), *Mesorhizobium* (3.36–29.12%), *Sinorhizobium* (2.28–23.55%), *Pseudomonas* (0.15–15.62%), and *Skermanella* (3.75–15.43%) (Figure 4b). The relative abundance of *Bradyrhizobium* was most predominant in organic farming practice under chickpea cropping. Moreover, *Pseudomonas* also showed higher relative abundance in organic treatment, whereas *Streptomyces* is enriched in soybean cropping with conventional farming soil.

## 3. Discussion

In the current study, organic farming practice stimulates TN, SOC, and TP [19]. Microbial biomass P (MBP) increases as a result of organic farming practice that stimulates the biological activity of soil in comparison to conventional farming. The organically amended soil is well established to increase organic matter in the fields [20]. Similar studies have shown an increase in organic matter content in organic farming and organic matter-rich soil to have long-term potential to sustain nutrient release [21,22]. The pH is increased in organic farming soil in comparison to the conventional counterpart. The lowering of pH in conventional farming soil maybe because of nitrification of NH^4+^ and thus H^+^ ion is produced which results in enhanced soil acidity. Chakraborty et al. showed a decrease in pH with increasing chemical fertilizer application [23]. In this study, the available P is significantly (*p* < 0.001) increased under the conventional farming practice. Similar results are also reported by Liu et al. [24]. Fraser et al. reported the same trend that indicated low available P in organic management [10]. This may be because fertilizer P administers orthophosphate usually amplifying labile inorganic P [25]. However, Sakurai et al. reported the opposite trend [13]. This discrepancy in available P maybe because of confounding features, including the quantity of phosphorous in fertilizers, crop P demand, and diverse agricultural practices.

Consistent with our hypothesis, the farming practices and crop types influenced the abundance and *phoD* gene bacterial communities. In the present investigation, organic farming stimulated the alkaline phosphatase activity of soil. Higher ALP activity was determined in organic farming than in the conventional one with significant differences (*p* < 0.001) (Figure 2a). It has been reported that organic matter application enhances the ALP activity in the soil [9,10,11,13]. The possible reason for the increased ALP activity in organic farming may be increased SOC that allows bacteria to proliferate due to an additional carbon source [6]. In addition, the amendment of cattle manure in soil results in a significant increase in alkaline phosphatase activity [26]. *phoD* gene copy number was significantly higher (*p* < 0.001) in soils of organic farming fields (Figure 2b). The earlier studies also reported similar results in the long-term manure-fertilized soil [8,9,10]. It is suggested that *phoD* abundance was highest in organic treatment with the highest alkaline phosphatase activity and SOC that increases organic matter suggesting higher nutrient content which accordingly increases bacterial abundance [27]. In organic farming practice, the organic fertilizer, low in available P content, and plenty of C-rich substrate possibly influenced the proliferation of various *phoD*-gene-containing bacterial population, hence increasing the *phoD* abundance and alkaline phosphatase activity [28]. In addition, we found a significant (*p* < 0.01) and strong positive correlation between OTUs, Shannon index, and Simpson index and alkaline phosphatase activity and *phoD* copy number (Table 2). The previous report indicated a significant positive correlation between *phoD* gene copy number and the bacterial community such as Shannon and Chao 1 diversity [18]. Zhu et al. showed a positive correlation between Chao 1 index and alkaline phosphatase activity, suggesting that *phoD*-gene-harboring bacteria may be highly activated while secreting ALP enzyme [17]. Similarly, a significant (*p* < 0.001) variation was observed among crop species for the activity of alkaline phosphatase and *phoD* abundance. The elevated ALP activity and the abundance of *phoD* bacteria exhibited a trend of maize > chickpea > mustard > soybean fields in soil samples (Figure 2a,b). This may be due to the influence of varied plant species on soil microbes that exhibit variability among plant physiological attributes including exudates of root [29] that possibly differ with crops and physiological attributes [30].

Recently, many studies have been reported demonstrating the shifts in *phoD*-containing bacterial population in soils with organic and inorganic fertilization [5,6,8,13,28]. As existing literature in the context of Indian agroecosystem suggests scarcity of knowledge about *phoD* encoding bacterial assemblages; however, agroecosystems situated in other countries have been analyzed for the same. Wei et al. studied that the P fertilization influences phosphorous mineralizing microbes in paddy soil, and all the sequences are classified into five classes: Alphaproteobacteria, Betaproteobacteria, Gammaproteobacteria, Cyanobacteria, and Actinobacteria [31]. The dominant genera of this study were *Methylobacterium*, *Methylomonas*, and *Bradyrhizobium.* Here, in this study, *phoD* taxonomic classification revealed that *Proteobacteria* and *Actinobacteria* were the two phyla among which *Proteobacteria* showed dominance in both farming practices. The investigations on *phoD* bacterial population identified similar genera [14,28]. However, *Proteobacteria* was the most dominant in organic farming samples. The previous study also detected *Proteobacteria* as the dominant phylum [6,8,28]. The dominance of phylum *Proteobacteria* may be because of the supplement of compost which contributed to an increased level of soil nutrients (e.g., SOC, TN, and TP) and stimulated the copiotrophic bacterial growth and resulted in a shift of *phoD*-harboring bacterial community. Tian et al. reported a higher abundance of *Proteobacteria* in swine manure added to soil with increased soil nutrients and promoted the proliferation of copiotrophic phyla, i.e., *Proteobacteria* [32]. We have also observed an increased level of *Actinobacteria* in conventional farming as compared to the organic amendment. *Actinobacteria* are more apparent to be an index for inorganic fertilization as compared to organic fertilization [33]. The genera *Ensifer* belonging to the phylum *Proteobacteria* was the most predominant genera and its relative abundance was highest in organic farming practice with soybean cropping. The previous studies on total and P-mineralizing bacterial communities in maize cropping detected similar genera *Ensifer* in soil fertilized with compost than chemical and nonfertilized soil [34].

The higher abundance of *Ensifer* sp. in soils managed with organic farming compared to the conventional suggests that organic fertilization perhaps enriched this genus in soil and accordingly strengthen their abundance in the rhizosphere [34]. The relative abundance of *Bradyrhizobium*, a free-living and nitrogen fixer that is present in all the samples, indicates that this genus perhaps plays a major role in linking nitrogen (N) and phosphorous (P) cycle [28]. Therefore, further study is imperative to acknowledge the role of *Bradyrhizobium* sp. in establishing the possible link between nitrogen cycling and phosphorous turnover under diverse conditions, which may contribute to accelerating ALP activity as well as phosphorous transport rates and also alter soil nitrogen pools [28]. In another study, Gitonga et al. [35] observed the effect of farm management (conventional and organic farming) system on *Bradyrhizobia* species abundance and diversity and reported proliferation of *Bradyrhizobium* in organic farming practice and hence enhanced *Bradyrhizobial* diversity. This may be because organic farming has been revealed to increase soil organic carbon which provides the required energy for microbes and thus increases their abundance and diversity as compared to conventional farming practice [36]. Here, in this study, we observed that organic farming stimulated the proliferation of *Pseudomonas* (relative abundance: 15.62%) in soils planted with chickpeas. Hu et al. also reported an increased relative abundance of *Pseudomonas* in the organic amendment [6]. It is reported that several species of *Pseudomonas* grow rapidly under straw as the only carbon source, on the basis of their plant lignin and hemicellulose degradation capacity [37]. Moreover, *Pseudomonas* sp. inhabits several P-mineralizing bacterial populations [38,39]. Another dominant genus identified in this study was *Streptomyces*. Hegyi et al. also reported *Streptomyces* as abundant, potential phosphate-solubilizing bacteria in soil and *Streptomyces liacinus* as a *phoD* gene encoding isolate [18]. It is established that apart from plant growth-promoting capabilities, *Streptomyces* species are generally associated with a phosphorous transformation, including phosphorous solubilization and mineralization [11,18]. Moreover, several *Streptomyces* species reported secreting alkaline phosphatase enzyme, namely, *S. griseus*, *S. hiroshimensis*, and *S. hygroscopicus* [40,41]. The organic farming practice showed the greater OTUs richness and Shannon diversity index compared to conventional farming (Table 1).

The observed OTUs were positively correlated with ALP activity and *phoD* abundance. These findings are in accordance with Sakurai et al. [13] showing that in organic matter amended soils *phoD* encoding bacterial assemblages shifted differently from those in conventional farming supplied with chemical fertilizers. Several earlier studies have also reported a change in *phoD* encoding bacterial assemblages in response to different fertilization management [11,12,42]. Our findings, as well as the previous reports, suggested that the change in *phoD* bacterial community by organic fertilizer might be caused by community composition turnover, i.e., existing OTUs replacement with the new OTUs. Moreover, organic farming showed higher alkaline phosphatase activity than conventional farming, and the variation in OTU composition of *phoD* may have enhanced enzymatic activity [42]. According to Watts et al. [43], the bacterial diversity in soil could be enhanced by the bacteria supplemented through the organic fertilizer.

Therefore, in the present study, we found that organic fertilizer incorporation strengthens the *phoD*-containing bacterial richness in comparison to conventional farming, suggesting a substantial effect of the introduced bacterial population from organic fertilizer on the alpha diversity of microbes. Our results revealed the effect of crops on the *phoD* bacterial population, but no particular trend was observed in test crops at the genera level. The OTU richness of *phoD* was highest in soils vegetated with maize compared to chickpea, mustard, and soybean field soils. Neal et al. have also reported that the effect of crop type on the phosphohydrolase genes was significant [44]. It is well-established fact that plants excrete a composite mixture of chemical compounds from the roots in the soil, which could possibly favor discrete microbial communities towards the rhizospheric zone [45,46,47]. Through enzymatic hydrolysis (phosphatase activity) of organic P and solubilization of mineral phosphate, these microbes can induce a supply of orthophosphate in soil [48].

## 4. Materials and Methods

### 4.1. Study Site and Experimental Design

The sampling sites were located at the agriculture field of Dagmagpur (Mirzapur district of Uttar Pradesh) (25°9′ N, 83°34′, 80 m above MSL), India. This zone has a seasonal tropical monsoonal climate with an average rainfall of 849.9 mm annually and the mean temperature (minimum to maximum) generally ranges between 8 °C and 10 °C in January and 38 °C and 42 °C in June. The soil of the study site was Alfisol with a silty sandy texture (32:64:4, sand:silt:clay). The agricultural farm was governed by the farmers, and the study site has a long (approximately 30 years) agricultural history. In this area, besides selected test crops for the present study, rice and wheat are the major crops. The soil total P was 152.17 µg g^−1^ in organic and 127.08 µg g^−1^ in conventional farming practice. The main soil physicochemical properties were given in [19]. 

Two agricultural fields having two different farming practices were selected: one field received compost (organic farming) and another field mineral fertilizer (conventional farming). The experimental plots were designed in randomized complete block design including three blocks (5 m × 4 m with 1 m gap) per site and a treatment combination of 4 crops × 2 farming practices. To avoid edge effects, the organic and conventional farming experimental plots were separated by a 100 m distance. In the organic farming plot, compost was added as an organic supplement. The compost applied is composed of crop residues and cow dung prepared by NADEP (Narayan Deorao Pandharipande) technique [49]. After maturation, the compost was dispersed manually at the rate of 15 tons ha^−1^ and plowed up to 15 cm depth to mix and homogenize before the cropping season of *Rabi* and *Kharif*. No other nutritional supplement was added apart from cow urine (1:50; urine:water dilution ratio), which is added twice, during the vegetative and flowering stages of the crops as a nitrogen source at the interval of 40 days. The cow urine used in the present study comprises 15 g N L^−1^. In conventional farming, as per the standard practice, NPK (chemical fertilizer) was applied at the rate of 120, 40, and 60 kg ha^−1^ in *Rabi* crops, and for the *Kharif* crops, the rate of NPK application was 20, 40, and 60 kg ha^−1^. This was executed twice annually, i.e., one-time application in both seasons as a basal dose. There were four crops selected in this study. Two each from *Rabi* (chickpea: *Cicer arietinum L.* var Pusa-256, and mustard: *Brassica campestris* var. T-151) and *Kharif* (maize: *Zea mays* var. Ganga II and soybean: *Glycine max* var. PS-1225). The experiments were conducted for two cropping seasons (2017–2019). No pesticides or fungicides were added, and weeds were removed manually.

### 4.2. Soil Sampling and Soil Physicochemical Analyses

Rhizosphere soil samples in triplicate (0–15 cm) from both (organic and conventional) farming fields were collected randomly at the mid-flowering stage of a crop growth cycle. Soil adhered to the rhizosphere zone was collected after tapping the root gently of each test crop in a plastic bag. Soil samples (in triplicate) were mixed and homogenized and sieved (2 mm mesh) to remove plant debris. The homogenized soil samples are divided into two parts: one part stored at −20 °C for downstream analyses of *phoD*-harboring bacterial community and qPCR experiments and the other kept (at 4 °C) for the analyses of ALP activity and soil properties. Soil physicochemical properties, alkaline phosphatase activity, and *phoD* gene abundance were measured for two consecutive years (2017–2019) in soil samples, and data were pooled as average data for two years and the *phoD* diversity analysis was performed only for second-year soil samples. All the basic physical and chemical properties of soil were analyzed following the standard protocols [50]. Total P was analyzed as per Allen et al. [51]. To measure soil available P, the method of Olsen et al. was followed [52]. Microbial biomass phosphorus (MBP) was measured following the standard protocol of Brookes et al. [53], and crop biomass was measured as per Neha et al. [19].

### 4.3. Assay of Soil Alkaline Phosphatase (ALP) Activity

Using *p*-nitrophenol phosphate (*p*-NPP) as substrate, ALP activity was measured [54]. The analysis was carried out by taking rhizosphere soil (1 g) with 1 mL modified universal buffer (pH 11), *p*-NPP, and incubating at 37 °C for 1 h. After 1 h, 0.5 M NaOH was mixed to terminate the reaction. The reaction mixture was filtered, and the *p*-nitrophenol (*p*-NP) was measured spectrophotometrically (at 420 nm). The activity was recorded as µmol of *p*-NP g^−1^ soil h^−1^. 

### 4.4. Soil DNA Extraction and phoD Gene Quantification

Total genomic soil DNA was extracted from 0.5 g frozen soil by FastDNA Spin Kit. NanoDrop 2000 spectrophotometer was used to measure DNA concentration and quality. The *phoD* gene copy number (abundance) was quantified by qPCR (iCycler iQ5 thermocycler; Bio-Rad, Hercules, CA, USA). The extracted DNA was amplified for *phoD* using ALPS F-730 and ALPS R-1101 primers as described by Sakurai et al. [13]. The PCR reaction mixture (20 µL) contains 10 µL of PowerUp^TM^ SYBR Green Master Mix, 0.5 µL of each primer concentration (10 µM), DNA template (2 µL), and water (nuclease-free) to make up the final volume to 20 µL. The PCR conditions for amplification of *phoD* gene were as follows: 3 min at 94 °C (initial denaturation), 40 cycles at 94 °C for 1 min (denaturation), at 61 °C for 45 s (annealing), and final extension at 72 °C for 45 s. Data were tested for PCR amplification efficiencies which were 114.5% and R^2^ = 0.963.

### 4.5. Illumina MiSeq High-Throughput Sequencing for phoD Gene Amplicons and Data Analysis

The Illumina Miseq 300 bp paired-end sequencing platform was used to assess the *phoD*-gene-containing bacterial population. The target gene (*pho D*) was amplified in rhizosphere soil DNA using ALPS-F730 and ALPS-R1101 primers [13]. The indexed paired-end library was prepared by adding Illumina Nextera XT compatible adapters to the forward and reverse primer sequences. The reaction mixture comprises template DNA (50 ng), KAPA Hifi HotStart Ready Mix (KAPA Biosystems, Wilmington, NC, USA), and modified primers (100 nM) ALPS-F730 and ALPS-R1101. PCR conditions were set as initial denaturation (at 94 °C for 3 min), followed by denaturation (30 cycles at 94 °C, 1 min), annealing (61 °C, 45 s), extension (72 °C, 30 s), and terminal extension (72 °C for 7 min). The amplicons were cleaned up and subjected to quantitate libraries using a quantitation assay of Qubit DNA high sensitivity (ThermoScientific, Grand Island, NE, USA). The quantity of the library was corroborated with the help of the D7500 DNA kit and Agilent Bioanalyzer (Agilent Technologies, Santa Clara, CA, USA). Illumina MiSeq 2 × 300 bp platform (Illumina, San Diego, CA, USA) was used for sequencing as per the manufacturer’s protocol. The generated raw targeted phoD gene amplicon sequences have been deposited to the NCBI sequence read archive (SRA) database under the BioProject accession number PRJNA797670.

### 4.6. Sequence Analysis

The sequences were analyzed as per Bhardwaj et al. [55] with slight modifications for the *phoD* gene. The FastQC was used to overview raw forward (R1) and reverse (R2) reads for basic quality control [56]. Raw sequences were quality filtered and trimmed using Trimmomatic V0.35 with criteria such as (i) adaptor sequences removal and (ii) eliminating unclear reads (reads having undefined nucleotides “N” > 5%) and low-quality sequences. The quality-passed (forward and reverse) joining of paired reads was executed by PEAR (Paired-End reAd mergeR), and the remaining single reads were discarded [57]. The joined reads with a quality score of < 30 and sequence size with less than 250 bp and more than 380 bp sequences were filtered out to obtain high-quality sequences (HQS). An appropriate pipeline Quantitative Insights Into Microbial Ecology (QIIME), version 1.9.0, was used for the analysis of high-quality reads [58] for bacterial diversity estimation. Initially, the quality-passed reads were screened against the funGene database regarding *phoD* sequences using the HMMER model with default settings. This allowed us to obtain only the *phoD*-gene-specific amplicon reads and filtered the unwanted reads such as 16S rRNA contamination and chimeric reads. Then, the *phoD*-gene-featured sequences were subjected to operational taxonomic units (OTUs) determination at 75% similarity clustering employing pick_otus.py script [59]. The OTUs were rarefied to the least library size (2629 reads) to find out the taxonomic diversity alpha indices, such as observed OTUs, Good’s coverage, Chao1, Simpson, Shannon, after discarding OTUs with <10 reads count. Furthermore, the taxonomic classification of reads was obtained using a Kaiju metagenome classifier with the k-mer setting of 31, integrated into MGX software.

### 4.7. Statistical Analyses

To study the effect of farming practices and crops on the physicochemical and microbiological properties of soil, MANOVA (multivariate analysis of variance) was conducted with Tukey’s post hoc test (*p* < 0.05). Pearson correlation test was used to study the relationship between diversity indices and soil parameters (IBM SPSS Statistics 20). Canonical correlation analysis (CCA) was employed to evaluate the effect of farming practices and crops to explore the correlation between soil variables and *phoD*-gene-harboring bacterial community using PAST v 3.20.

## 5. Conclusions

The farming practices and crop types change the composition of *phoD*-containing bacterial communities. The present study suggests that alkaline phosphatase activity, *phoD* abundance, and OTUs richness were increased in response to organic farming practice. *Rhizobiales* showed dominance at the order level. *Bradyrhizobium*, *Ensifer*, *Streptomyces*, and *Pseudomonas* were detected as the dominant members. This study will provide a better understanding of the significance of the alkaline phosphatase enzyme and its role in P mineralization to improve P management strategy to maintain sustainable agriculture. Future research is needed to address the influence of various types and quantities of fertilizers under different cropping systems on *phoD* communities for stimulating P availability in agricultural soils.

## Figures and Tables

**Table 1:** Diversity indices (rarefied at minimum library size, i.e., 2629 reads).

Crop Type	Farming Practice	Observed OTUs	Chao 1	Shannon	Simpson	Good’s Coverage
Chickpea	Organic	492	768	7.7	1.0	0.9
	Conventional	477	805	7.0	1.0	0.9
Mustard	Organic	396	612	6.4	1.0	0.9
	Conventional	383	825	6.1	0.9	0.9
Soybean	Organic	376	598	6.6	1.0	0.9
	Conventional	346	540	6.1	0.9	0.9
Maize	Organic	672	1121	8.1	1.0	0.9
	Conventional	515	736	7.6	1.0	0.9

**Table 2:** Pearson correlation coefficient between diversity indices and soil parameters in different crops and farming practices (* *p* < 0.05, ** *p* < 0.01). ALP, alkaline phosphatase; MBP, microbial biomass P.

	pH	Available P	ALP Activity	MBP	Mineral N	*phoD* Gene Copy Number	Root Biomass	Shoot Biomass
Observed OTUs	0.23	−0.46	0.840 **	0.790 *	0.22	0.885 **	0.90 **	0.88 **
Chao1	0.08	−0.29	0.59	0.64	0.23	0.68	0.861 **	0.69
Shannon Index	0.23	−0.53	0.906 **	0.815 *	0.26	0.935 **	0.78 *	0.81 *
Simpson Index	0.27	−0.58	0.867 **	0.795 *	0.21	0.879 **	0.67	0.63
Good’s coverage	−0.15	0.34	−0.64	−0.67	−0.18	−0.725 *	− 0.894 **	−0.747 *

## Data Availability

The *phoD* gene amplicon sequences have been deposited to the NCBI sequence read archive (SRA) database under the BioProject accession number PRJNA797670.
